# Log file‐based quality assurance method for respiratory gating system

**DOI:** 10.1002/acm2.70101

**Published:** 2025-04-08

**Authors:** Wonjoong Cheon, Young Kyu Lee, Yunji Seol, Chan‐beom Park, Hong Qi Tan, Kyu Hye Choi, Young‐nam Kang, Byung Ock Choi

**Affiliations:** ^1^ Department of Radiation Oncology Seoul St. Mary's Hospital College of Medicine The Catholic University of Korea Seoul Republic of Korea; ^2^ Department of Radiation Oncology Seoul St. Mary's Hospital The Catholic University of Korea Seoul Republic of Korea; ^3^ Department of Biomedicine & Helath Sciences College of Medicine The Catholic University of Korea Seoul Republic of Korea; ^4^ Division of Radiation Oncology National Cancer Centre Singapore Singapore Singapore; ^5^ Oncology Academic Clinical Programme Duke‐NUS Medical School Singapore Singapore; ^6^ School of Physics and Mathematical Science Nanyang Technological University Singapore Singapore

**Keywords:** Log file‐based analysis, quality assurance (QA), radiation therapy, respiratory gating, RGSC (Respiratory Gating for Scanner) system

## Abstract

**Background:**

As medical linear accelerator technology advances, enabling higher dose rate deliveries, hypofractionation regimens has increased. This necessitates respiratory gating systems that synchronize radiation delivery with tumor position, requiring simple rigorous quality assurance (QA) to ensure treatment accuracy and patient safety.

**Purpose:**

This study aimed to propose log‐based QA for respiratory‐gated radiation therapy using the respiratory gating system and treatment machine.

**Methods:**

4D CT scans were performed with a Varian motion phantom using a Varian Respiratory Gating for Scanner (RGSC). A treatment plan using 25%–75% respiratory phases with 100 MU was created and delivered to a solid water phantom. Treatment logs containing respiratory signals, beam on/off flags, and frame information were extracted from the treatment planning system's offline review. Log file analyses were conducted using in‐house softwares to assess temporal synchronization between respiratory phases and beam triggers. Output measurements using a calibrated ion chamber (FC65G) were performed to evaluate dosimetric accuracy. Additionally, EPID images were acquired in cine mode and analyzed frame‐by‐frame to independently verify beam delivery timing.

**Results:**

Log file analysis revealed precise temporal synchronization, with mean time differences of 0.03 s ± 0.05 s between the planned 25% phase and beam‐on, and −0.04 s ± 0.05 s between 75% phase and beam‐off. The log‐derived beam‐on duration (2.61 s ± 0.02 s) closely matched the planned duration (2.66 s ± 0.00 s). Three‐month log data showed consistent temporal accuracy, with trigger‐on times remaining stable at 2.60 s ± 0.01 s across all measurements. Supporting ion chamber measurements confirmed dosimetric agreement between gating and non‐gating modes (difference: 0.05 cGy ± 0.09 cGy)

**Conclusions:**

The proposed log file‐based QA method demonstrated high accuracy and reproducibility in assessing respiratory gating performance. This approach provides an efficient, objective method for standardizing QA procedures in respiratory‐gated radiation therapy, enhancing treatment accuracy and patient safety.

## 1 INTRODUCTION

Managing physiological motion during radiation therapy is crucial to ensure precise dose delivery to the target tumor while minimizing radiation exposure to surrounding healthy tissues. Tumor movement due to various physiological processes such as breathing, cardiac motion, and swallowing can result in unintended dose to non‐targeted organs, potentially leading to suboptimal treatment outcomes and an increased risk of complications and side effects.^1^, ^2^, ^3^, ^4^ Among these motion sources, respiratory motion presents particular challenges due to its amplitude and frequency. To address these respiratory‐induced challenges, respiratory gating systems have been developed to synchronize radiation delivery with the tumor's position during the patient's respiratory cycle, beam on and off based on the tumor's motion.

Advancements in radiation therapy technology have facilitated higher dose rate deliveries and the implementation of hypofractionated treatment regimens. These advancements hold the promise of improved treatment efficacy and reduced overall treatment duration. Accordingly, the importance of quality assurance (QA) for guided radiation therapy systems has been increasingly emphasized to maintain high treatment accuracy and safety, particularly in managing respiratory motion.

QA for respiratory gating systems involves verifying that the system accurately tracks respiratory motion and synchronizes beam delivery with the planned respiratory phases. Reports and studies, including those by Task Group 76a (TG‐76a) of the American Association of Physicists in Medicine (AAPM),^4^ emphasize comprehensive QA for respiratory motion management. With the increasing adoption of hypofractionation regimens and advanced delivery techniques, TG‐76a guidelines particularly address interplay effects and recommend stricter motion management criteria for SBRT treatments compared to the 5 mm threshold in the original TG‐76. The TG‐142, and TG‐147 guidelines provide detailed respiratory gating system QA information for medical linear accelerators, including QAs for beam output consistency, temporal accuracy of gating, routine checks, and rigorous baseline establishment to ensure optimal system performance.^5^, ^6^

Ensuring the reliability of respiratory‐gated radiation therapy requires a clear definition and measurement of the delay time within the respiratory gating system. This delay encompasses the delay from the external surrogate signal (e.g., from a charge‐coupled device or an infrared camera), the delay in the beam trigger (on/off), and the delay for the beam to reach the measurement device.

Previous researchers have employed dosimetric methods to verify the accuracy of respiratory gating systems using phantoms with distinct density differences undergoing periodic motion. Techniques such as using an electronic portal imaging device (EPID),^7^, ^8^, ^9^, ^10^ a two‐dimensional ion chamber array detector,^11^, ^12^ radiochromic film,^13^, ^14^ and optically stimulated luminescent dosimeters^15^ have been utilized to evaluate the accuracy of respiratory‐guided systems. However, these dosimetric methods require devices capable of precise periodic motion, and the spatial and temporal resolution of the dosimetry devices can affect the measurement results.

**FIGURE 1:**
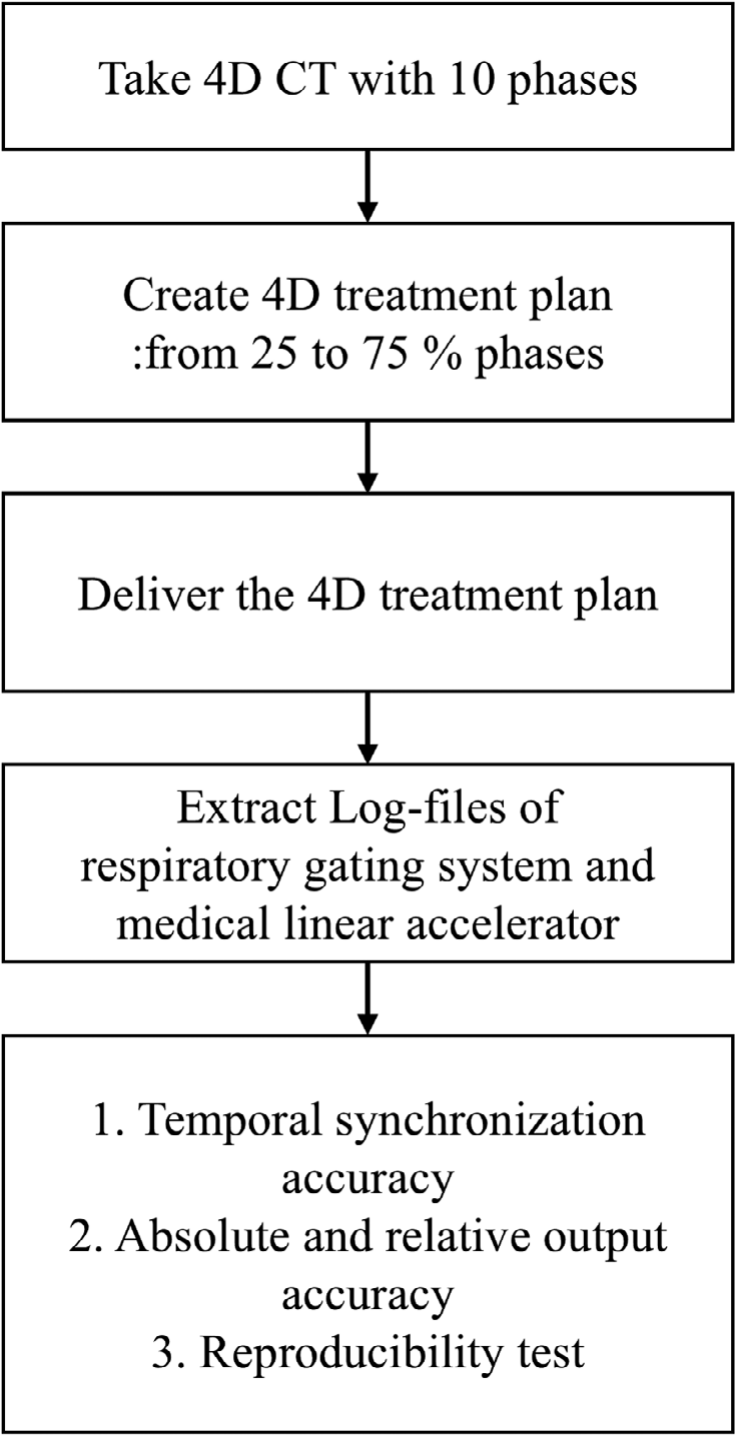
Study diagram for log‐based quality assurance for respiratory‐guided radiation therapy.

In this study, we propose a log file‐based QA method for respiratory gating systems used with medical linear accelerators. This approach could offer a detailed assessment of the system's accuracy and contribute to the development of more efficient and accurate QA procedures for respiratory‐gated radiation therapy (Figure 1).

## 2 MATERIALS AND METHODS

### 2.1 4D CT imaging and treatment planning

In our institution, a SOMATOM go.Open Pro (Siemens, Erlangen, Germany) four‐dimensional (4D) computed tomography (CT) scanner was installed with the Varian Respiratory Gating for Scanner (RGSC; Varian Medical Systems, Palo Alto, CA, USA). The RGSC system was located in both the CT simulation room (for scanner) and the treatment room (for respiratory gating system), where TrueBeam (Varian Medical Systems, Palo Alto, CA, USA) was installed. The RGSC was properly calibrated according to the vendor‐provided documents. The system achieved spatial localization accuracy within 2.0 mm.^16^

4D‐CT scan images were acquired by placing a reflector block on the Varian motion phantom, which underwent periodic motion. The Direct i4D technique was used for this acquisition.

Treatment plans were created with the following parameters: treatment machine of TrueBeam, gantry angle of 0.0°, collimator angle of 0.0°, couch angle of 0.0°, and the multileaf collimator (MLC) set to an open configuration. The field size was 10.0 cm × 10.0 cm. The respiratory gating system's breathing predictive filter was set to 0, which disables beam interruption due to breathing irregularities, making it suitable for controlled experimental setups with regular motion patterns. The treatment plans utilized respiratory phases ranging from 25% to 75% of the respiratory cycle. A planned monitor unit (MU) of 100.0 MU was used. Additionally, EPID image acquisition was configured for radiation field measurement and verification.

### 2.2 Experimental setup

**FIGURE 2:**
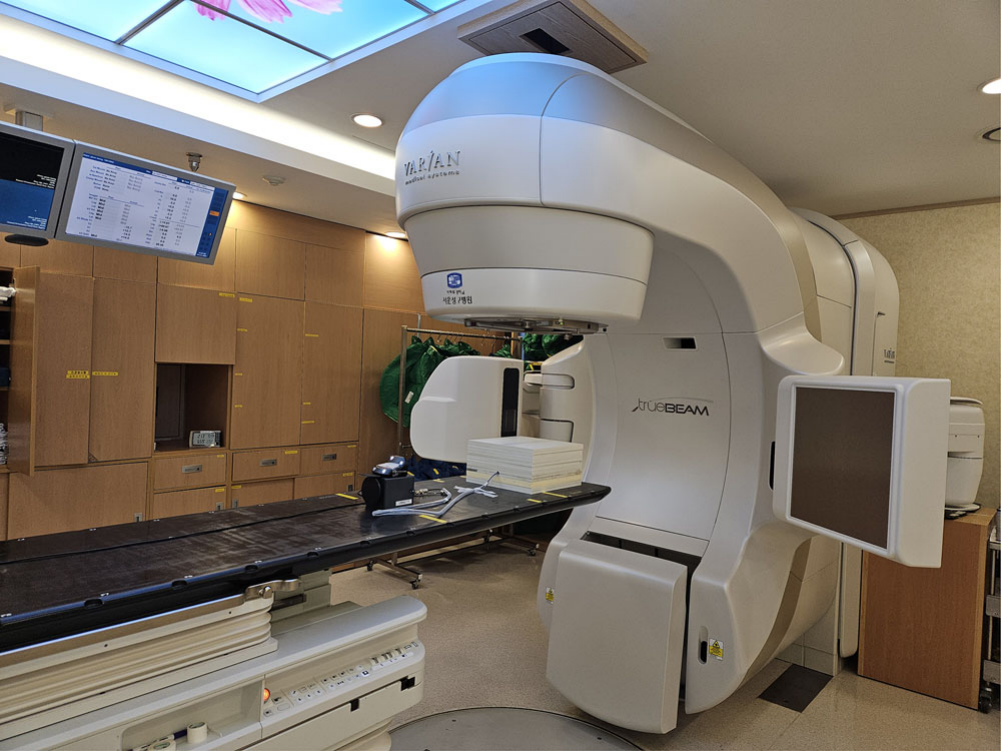
Experimental setup for log‐based respiratory gating system quality assurance showing the Varian motion phantom with reflector block and solid water slab phantoms in the treatment room.

To ensure both temporal accuracy between the planned phase times and the beam on/off triggers and output consistency with the respiratory gating mode, the Varian motion phantom was placed at a safe distance from the radiation field on the couch top, with a reflector block positioned on the phantom to simulate respiratory motion (Figure 2). Additionally, a solid water phantom (30.0 cm × 30.0 cm × 10.0 cm) was placed at the isocenter with a source‐to‐surface distance of 100.0 cm, and another solid water phantom (30.0 cm × 30.0 cm × 5.0 cm) was placed beneath the 10.0 cm phantom to provide backscatter. Dose measurements were conducted using an ion chamber (FC65G, Ion Beam Application, Belgium), which was placed at a depth of 10.0 cm in the phantom and a DOSE1 electrometer (Scanditronix Wellhofer, Sweden), both calibrated by an accredited institution in the Republic of Korea. Measurements were performed using the standard monthly medical linear accelerator output test geometry. The reference absolute point dose was measured in a non‐respiratory guided radiation therapy mode with 100.0 MU.

### 2.3 Radiation delivery with the respiratory gating system

The respiratory gating system continuously monitored the periodic motion of the phantom and synchronized radiation delivery with the planned respiratory phases in real time. The planned MU was delivered using the phase gating mode. Treatment logs were extracted from the offline review in the Eclipse treatment planning system (Varian Medical Systems, Palo Alto, CA, USA). These logs contained detailed data, including respiratory signals representing tumor motion in three orthogonal axes (x, y, and z), beam on/off flags, and frame information (corresponding time for each frame, etc.) of the movie recorded by EPID.

### 2.4 Temporal synchronization analysis

The data analysis aimed to evaluate the synchronization between the respiratory signals and the beam on/off triggers at the planned respiratory phases. MATLAB (R2023a, Update 6, License: 821902) was used to develope a in‐house software for the treatment log file analysis. Among the signals from the respiratory gating system, the signal for anterior‐posterior (AP) motion was extracted and periodically divided. The AP motion data were Min‐Max normalized and smoothed using the “movmean” method with a five‐pixel window length. Then, the start and end points of each respiratory cycle were analyzed, and the mean and standard deviation of the duration between the 25% and 75% phases were calculated. The accumulated signal was used to generate an average periodic signal.

The beam on/off trigger signal was analyzed to identify the duration of each trigger‐on time. The durations of the beam‐on periods were calculated by measuring the time differences between corresponding on and off triggers. We compared the time durations for the 25% to 75% phases from the respiratory signals with the trigger‐on times. In addition, for each respiratory period, the time differences between the 25% phase and the beam‐on trigger, as well as the 75% phase and the beam‐off trigger, were calculated.

### 2.5 EPID‐based verification of beam delivery

To independently verify the temporal accuracy of the respiratory gating system, EPID measurements were performed during phantom irradiation. The EPID was configured to acquire images in cine mode with a frame rate of approximately 25 frames per second (time resolution of 0.04 s). A 5.0 cm × 5.0 cm region of interest (ROI) centered on the beam central axis was established for quantitative analysis.

The acquired EPID images were exported in RT IMAGE format for frame‐by‐frame analysis. Image processing and analysis were performed using Python (version 3.10) with specialized libraries including pydicom (version 3.0.1), imageio‐ffmpeg (version 0.6.0), and opencv‐python (version 4.11.0.86), with additional post‐processing for noise reduction.

For each frame, the mean pixel intensity within the ROI was calculated and normalized to the maximum intensity to determine beam‐on periods. A threshold of 50% of the maximum pixel intensity was used to define beam‐on status. The beam‐on duration was calculated by multiplying the number of frames above this threshold by the frame time.

### 2.6 Reproducibility test

The reproducibility test aimed to evaluate the consistency and reliability of the log file‐based QA method for the respiratory gating system over 3 months. Tests were conducted at 1‐month intervals using the same experimental setup with the Varian motion phantom and solid water phantoms. The same ion chamber and electrometer were used, and the same treatment plan was applied each time with the same medical linear accelerator. Prior to each measurement, the medical linear accelerator's output was calibrated to deliver 1.0 cGy/1.0 MU according to the in‐hospital protocol based on the TG‐51.

The respiratory gating system monitored the Varian motion phantom's motion, and radiation was delivered in gated mode when the phantom's motion was within the planned 25%–75% respiratory phase window. The logs were extracted for analysis. Each measurement included data from 5∼6 respiratory cycles, and the standard deviation was calculated based on these cycles. This approach allowed us to assess both the immediate accuracy of individual measurements and the long‐term reproducibility of the respiratory gating system's performance with one measurement performed per month.

## 3 RESULTS

### 3.1 Temporal synchronization accuracy

The mean and standard deviation of the time duration from the 25% to 75% phases of the respiratory gating signal for each period were 2.66 s ± 0.00 s. For the beam status, the mean and standard deviation of the trigger‐on‐time for each period were 2.61 s ± 0.02 s. The time difference between these two measurements was 0.05 s ± 0.02 s.

**FIGURE 3:**
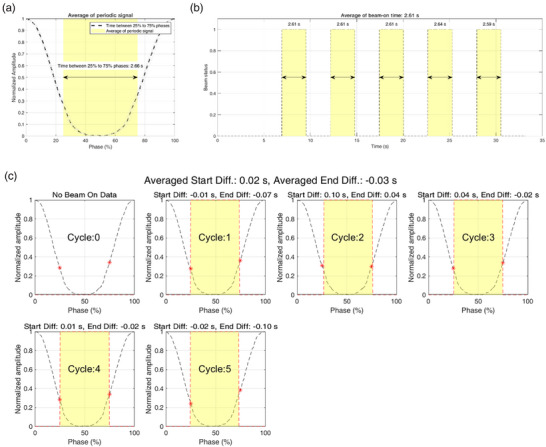
(a) Average periodic signal indicating the planned phase interval from 25% to 75%. (b) Trigger‐on times extracted from the treatment log file. (c) periodic analysis for respiratory gating treatment (red star: 25%, 75% phase, red line: trigger on/off).

The comparison of the time difference at the 25% and 75% phases of the respiratory gating signals with the actual beam on/off trigger times per period showed the following results: The mean time difference between the planned 25% phase start and the beam on the trigger was 0.03 s ± 0.05 s. The median time difference was 0.03 s. Similarly, the mean time difference between the planned 75% phase end and the beam‐off trigger was −0.04 s ± 0.05 s. The median time difference was −0.02 s. Figure 3 illustrates a representative example of a single measurement, showing the respiratory gating signal and the beam on/off trigger responses.

### 3.2 Absolute output accuracy

The reference point dose measurements obtained from the ion chamber during non‐respiratory gating treatment were compared with those obtained during respiratory gating treatment in the experimental setup. The mean and standard deviation of the reference point dose were 99.93 cGy ± 0.10 cGy. With respiratory gating mode, the mean and standard deviation of the measured point dose were 99.97 cGy ± 0.11 cGy. The difference was 0.05 cGy ± 0.09 cGy. These standard deviations demonstrate high measurement precision within a single session, showing variations well below 0.5%.

Over a 3‐month reproducibility test period, dose measurements showed consistent results. The mean and standard deviation of the point dose measurements during respiratory gating treatment were 99.93 cGy ± 0.06 cGy, 99.88 cGy ± 0.03 cGy, and 100.10 cGy ± 0.03 cGy for each month respectively.

### 3.3 EPID‐based verification results

Frame‐by‐frame analysis of the EPID cine images showed defined beam‐on periods that corresponded to the planned gating window. The mean beam‐on time for each respiratory period measured using EPID with respiratory gating mode was 2.64 s ± 0.06 s.

When comparing the EPID measurements with the log file analysis, the time difference between these two measurements was 0.01 s ± 0.06 s. This difference can be attributed to several factors including beam‐to‐EPID delay time, signal persistence in the EPID detector, and the limited time resolution of the EPID cine mode.

### 3.4 Reproducibility test

The reproducibility test over 3 months showed consistent results for temporal synchronization accuracy. The mean and standard deviation of the time duration between the 25% and 75% phases of the respiratory gating signal were 2.66 s ± 0.01 s, 2.66 s ± 0.01 s, and 2.67 s ± 0.01 s for each month respectively.

For the beam status activated by the trigger signal, the mean and standard deviation of the trigger‐on time for each period were 2.60 s ± 0.01 s, 2.60 s ± 0.01 s, and 2.59 s ± 0.01 s for each month respectively. These results demonstrate the consistent periodic behavior of the respiratory gating signal and the beam on/off triggers over the 3 months. The detailed results are tabulated in Table 1.

**TABLE 1:** Comparison of time differences between trigger on/off and planned phase per period: 3‐month measurement results.

		Cycles								
		1	2	3	4	5	6	AVG.	STD.	Median
Trial 1	Difference between 25% and trigger‐on (s)	−0.01	0.10	0.04	0.01	−0.02	N/A	0.02	0.04	0.01
	Difference between 75% and trigger‐off (s)	−0.07	0.04	−0.02	−0.02	−0.10	N/A	−0.03	0.05	−0.02
Trial 2	Difference between 25% and trigger‐on (s)	0.07	−0.01	0.04	0.01	0.04	0.10	0.04	0.04	0.04
	Difference between 75% and trigger‐off (s)	0.04	−0.07	−0.02	−0.04	−0.02	0.04	−0.01	0.04	−0.02
Trial 3	Difference between 25% and trigger‐on (s)	0.08	−0.04	0.03	−0.04	−0.01	0.10	0.02	0.05	0.01
	Difference between 75% and trigger‐off (s)	0.00	−0.14	−0.05	−0.11	−0.11	0.04	−0.06	0.06	−0.08

Additionally, the independent EPID‐based measurements conducted during the same periods showed comparable consistency, with mean beam‐on times of 2.69 s ± 0.05 s, 2.60 s ± 0.06 s, and 2.64  s± 0.03 s for each month respectively, further validating the stability of the respiratory gating system performance over time.

## 4 DISCUSSION

This study evaluated a log file‐based QA method for respiratory‐gated radiation therapy using the Varian treatment machine and respiratory gating system. Our log‐based measurements showed excellent agreement with conventional dosimetric measurements.

The temporal analysis revealed precise synchronization between respiratory phases and beam delivery with mean differences of 0.03 s ± 0.05 s at beam‐on and −0.04 s ± 0.05 s at beam‐off. Single‐session measurements demonstrated high precision with standard deviations of 0.10 cGy and 0.11 cGy for non‐gated and gated delivery respectively, well within the clinically acceptable range of 0.5%. The difference between gated and non‐gated delivery was only 0.05 cGy ± 0.09 cGy, demonstrating excellent dosimetric accuracy. The periodic analysis illustrated in Figure 3 further confirmed this precise temporal synchronization, and these comprehensive evaluations validated that our log‐based QA approach provides reliable verification of respiratory‐gated radiation therapy performance. This consistent accuracy in beam delivery timing suggests that the respiratory gating system effectively manages respiratory motion through its integrated prediction algorithm.^17^

These results align with previous studies emphasizing the importance of precise timing in respiratory‐gated systems.^1^, ^4^, ^18^, ^19^ The high precision observed corroborates findings from other investigations reporting similar accuracy levels in beam‐on and beam‐off delays under varying conditions.^10^

The high level of synchronization achieved between the respiratory gating system and treatment machine has significant implications for patient safety and treatment efficacy, particularly in hypofractionated regimens. The log file‐based QA method provides a detailed assessment of system accuracy and facilitates standardized QA procedures, which are essential for consistent performance across clinical settings.

This study presents several notable strengths in advancing respiratory‐gated radiation therapy QA procedures. The proposed log file‐based method offers a streamlined approach that simplifies the QA process. Logs can be used independently for efficient daily QA, while the combination of log analysis with ion chamber measurements enables more comprehensive monthly QA assessments. Additionally, the integration of logs with EPID verification provides an alternative daily QA option that balances efficiency with thoroughness. A critical strength of our approach is the ability to analyze precise starting and ending points of beam delivery relative to respiratory phases, detecting systematic timing errors that would be missed by conventional accumulated dosimetry methods. This temporal synchronization analysis reveals phase‐specific delivery discrepancies that impact treatment accuracy. Another key advantage of this method is its capability for post‐treatment analysis, allowing retrospective review of system performance without requiring additional patient or machine time. This flexible, multi‐tiered approach has been thoroughly validated through comprehensive 3‐month reproducibility testing, demonstrating consistent performance over time while enhancing the overall efficiency of system‐level QA procedures.

However, several limitations should be acknowledged. The study primarily relied on a specific phantom and controlled setup, which may not fully capture the complexity and variability of patient‐specific respiratory patterns encountered in clinical settings. A significant limitation inherent to log‐based QA is its inability to detect scenarios where a trigger signal is recorded but the beam fails to deliver due to hardware issues. While the combination of EPID and log analysis can help identify such discrepancies, this requires additional verification steps beyond basic log analysis. Furthermore, our validation focused on regular breathing patterns, while irregular patterns with various predictive filter size and baseline drift commonly seen in patients present additional challenges that would benefit from further investigation.

These limitations point to several important directions for future research. While our current method primarily serves as a system‐level QA tool for verifying the respiratory gating functionality, it has potential applications in patient‐specific QA workflows. Future studies should investigate implementing log‐based analysis alongside conventional patient‐specific QA, particularly focusing on variable respiratory patterns and irregular breathing that are common in clinical scenarios. Integration with emerging technologies such as surface guidance systems and artificial intelligence‐based motion prediction algorithms could further extend this method's capabilities, potentially enabling adaptive respiratory gating strategies based on historical patient‐specific log data patterns.

In summary, our log file‐based QA method for respiratory gating systems in medical linear accelerators demonstrates high accuracy and efficiency in assessing system performance. This approach provides a reliable foundation for system‐level QA procedures in respiratory‐gated radiation therapy, with the potential for expansion into patient‐specific applications through future technological integration.

## 5 CONCLUSIONS

This study presents a novel log file‐based QA method for respiratory‐gated radiation therapy, demonstrating high precision in synchronizing radiation delivery with respiratory phases. Our findings reveal high temporal accuracy between planned respiratory phases and beam triggers, excellent dosimetric consistency between gated and non‐gated delivery, and robust reproducibility over 3 months. The method's versatility allows for various implementation levels, from standalone log analysis for daily QA to comprehensive evaluations combining logs with dosimetric measurements for monthly QA. This approach streamlines the QA process while maintaining rigorous standards, showing significant potential for standardizing procedures across clinical settings. These results underscore the importance of efficient yet thorough QA protocols in ensuring treatment accuracy in respiratory‐gated radiation therapy, ultimately contributing to enhanced patient safety.

## AUTHOR CONTRIBUTIONS

Wonjoong Cheon involved in conceptualization, methodology, investigation, data analysis, and writing original manuscript. Young Kyu Lee and Yunji Seol involved in conceptualization and reviewing the manuscript. Chan‐beom Park, Hong Qi Tan, Kyu Hye Choi involved in methodology, investigation. Young‐nam Kang, Byung Ock Choi reviewing the manuscript.

## CONFLICT OF INTEREST STATEMENT

The authors declare no conflicts of interest.

## Data Availability

The data that support the findings of this study are available from the corresponding author upon reasonable request.
